# Association between Temporal Muscle Thickness and Overall Survival in Non-Small Cell Lung Cancer Patients with Brain Metastasis

**DOI:** 10.3390/curroncol29090508

**Published:** 2022-09-08

**Authors:** Young Il Kim, Ja Young Shin, Seung Ho Yang, Hyun Ho Kim, Byoung Yong Shim, Stephen Ahn

**Affiliations:** 1 Department of Neurosurgery, St. Vincent’s Hospital, College of Medicine, The Catholic University of Korea, Seoul 16247, Korea; 2 Division of Medical Oncology, Department of Internal Medicine, St. Vincent’s Hospital, College of Medicine, The Catholic University of Korea, Seoul 16247, Korea; 3 Department of Neurosurgery, Seoul St. Mary’s Hospital, College of Medicine, The Catholic University of Korea, Seoul 06591, Korea

**Keywords:** sarcopenia, temporal muscle, neoplasm metastases, brain neoplasm, lung neoplasm, survival analysis

## Abstract

Temporal muscle thickness (TMT) has recently been suggested as a novel biomarker of sarcopenia in head and neck malignancies. However, few studies have evaluated TMT as a prognostic marker in patients with brain metastasis. This study investigated the association of TMT with overall survival (OS) in non-small cell lung cancer (NSCLC) patients with brain metastasis. The records of all NSCLC patients with brain metastasis between 2009 and 2018 at St. Vincent’s Hospital were reviewed retrospectively. A total of 221 patients met our eligibility criteria. In the group with TMT thicker than the median, OS was longer than the group with TMT thinner than the median (240 days versus 139 days, *p* = 0.014). In multivariate analysis, the thicker TMT group had longer survival (HR 0.73 CI 0.56–0.96, *p* = 0.024). Male (HR 1.58 CI 1.19–2.09, *p* = 0.002) and older age (≥65 years) (HR 2.05 CI 1.53–2.74, *p* < 0.001) also showed statistical significance. We also performed subgroup analysis in older patients (≥65 years). In this subgroup of 107 patients, the thicker TMT group also showed longer OS than the thinner TMT group (209 days versus 82 days, *p* = 0.009). Our findings suggest that TMT can be a useful biomarker for OS in NSCLC patients with brain metastasis.

## 1. Introduction

Metastatic brain tumors are the most common type of intracranial malignancy [1]. Lung cancer accounts for the largest proportion of primary cancers among metastatic brain tumors, with non-small cell lung cancer (NSCLC) constituting the primary tumor type for approximately 50% of patients with brain metastasis [2]. Despite the development of various treatment modalities, median survival is approximately 7 months from the time of initial treatment of brain metastasis [3].

Sarcopenia is associated with prognosis in various cancer types [3,4,5,6,7]. Many studies have been conducted on sarcopenia in cancer patients since Mourtzakiw et al. reported that sarcopenia can be effectively diagnosed by measuring the L3 level of muscle volume on computed tomography (CT) [8]. Ranganathan et al. [9] and Leitner et al. [10] found a high correlation between temporal muscle thickness (TMT) and lumbar skeletal muscle cross-sectional areas in patients with brain tumors. Recently, several reports have suggested that TMT is representative of sarcopenia in various brain tumors and is related to prognosis [10,11,12,13,14,15,16,17,18]. However, research on brain metastasis is still lacking.

This study was conducted to validate previous study reporting a relationship between TMT and survival of patients with brain metastases in a routine clinical setting [14]. In contrast to previous studies, we performed a subgroup analysis of elderly patients (≥65 years) because muscle thickness correlates with age. In previous studies, there were no subgroup analyses of elderly patients to investigate the possibility that TMT could be a strong prognostic factor along with age.

## 2. Materials and Methods

### 2.1. Study Population

Our retrospective study was approved by the institutional review board of St. Vincent’s Hospital. The electronic medical records of NSCLC patients who were diagnosed with brain metastasis at our institution between 2009 and 2018 were examined. Our inclusion criteria were (1) NSCLC pathologically confirmed at primary sites or other sites of any metastasis, (2) brain metastasis confirmed on magnetic resonance imaging (MRI) by neuro-radiologists, or confirmed pathologically by neuropathologists. Our exclusion criteria were (1) non-accessible baseline clinical variates including temporal muscle thickness (2) no available survival data (3) any primary brain tumor before the diagnosis of brain metastasis.

### 2.2. Temporal Muscle Thickness

**Figure 1:**
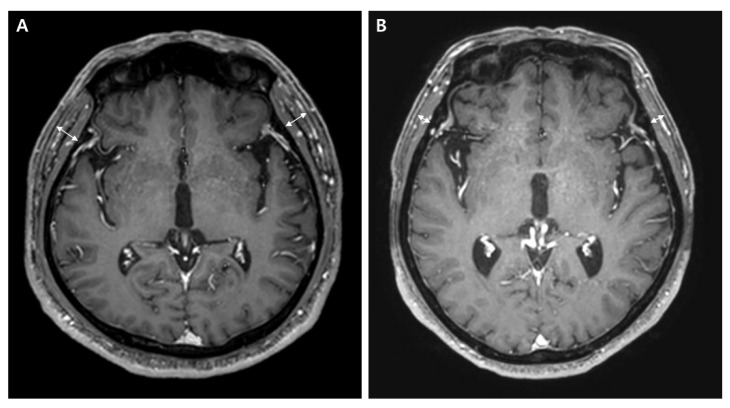
Representative cases of TMT measurements on brain MRI. (**A**) A 72-year-old male patient with an overall survival of 7 months (right TMT = 10.6 mm, left TMT = 11 mm, mean TMT = 10.8 mm) and (**B**) a 74-year-old male patient with an overall survival of 4 months (right TMT = 5.5 mm, left TMT = 6.2 mm, mean TMT = 5.9 mm).

TMT was measured on an axial 1 mm slice contrast-enhanced T1-weighted MRI performed at the initial diagnosis of brain metastasis. The axial MRI plane was oriented parallel to the anterior commissure-posterior commissure line. The perpendicular line to the long axis of the temporal muscle by using Sylvian fissure (anterior-posterior) and orbital roof (cranio-caudal) as anatomical landmarks. The TMT of the left and right side was summed and divided by two to yield the mean TMT. Representative examples of TMT measurement are illustrated in Figure 1. We set the cut-off value of TMT as the median TMT of the included patients. As there was a significant difference in TMT between male and female patients, we set different cut-off values. According to the cut-off value of TMT, the “thicker TMT group” was composed of patients who had a TMT thicker than the median, while the “thinner TMT group” was composed of patients who had a TMT thinner than the median.

### 2.3. Clinical Variates

The clinical variates include sex, age, pathological diagnosis, treatment modalities that the patients received after brain metastasis diagnosis, radiological findings of brain metastasis, performance score at the time of brain metastasis, and survival status and/or death date. After brain metastases were diagnosed, local treatment included radiation therapy, radiosurgery, and/or surgical resection. Through multidisciplinary discussion, the best modality for the patients was chosen considering the characteristics of brain metastasis and patient condition. Performance status was estimated according to the Eastern Cooperative Oncology Group (ECOG) scale. The presence of epidermal growth factor receptor (EGFR) gene mutation was evaluated by polymerase chain reaction. The presence of anaplastic lymphoma kinase (ALK) gene mutation was evaluated by immunohistochemistry staining. Survival status and/or death date were collected using the Korea Central Cancer Registry database.

### 2.4. Statistical Analysis

We calculated overall survival (OS) using days from the diagnosis date of brain metastasis by an MRI to the death date. Alive patients on 31 December 2021 were censored. All clinical variates were characterized in a descriptive manner. The differences in clinical variates between the thicker and thinner groups were evaluated using Chi-square test or Fisher’s exact test after testing normality for continuous variates. Kaplan–Meier survival curve analysis and the log-rank test were conducted to obtain the median OS of the groups. Univariate and multivariate analyses were performed using a Cox proportional regression model. Hazard ratios (HRs) and 95% confidence intervals (CIs) were calculated. Multivariate analysis was performed on variates with *p* values < 0.2, and *p* values < 0.05 were considered to indicate statistical significance. All statistical analyses were conducted using the RStudio, version 1.4.1717 (250 Northern Ave, Boston, MA 02210, USA).

## 3. Results

### 3.1. Patient Characteristics

Among the total of 221 patients who met the eligibility criteria, 112 patients were categorized into the “thicker TMT group” and 109 patients in the “thinner TMT group”. The cut-off value (medians) for male and female patients is 7.7 mm and 7.0 mm, respectively. The median TMTs of thicker group in male and female patients were 9.6 mm (range 7.7–14.8) and 8.3 mm (range 7.0–11.7), while the median TMTs of thinner group in male or female patients were 6.0 mm (range 2.4–7.6) and 5.7 mm (2.4–6.9), respectively. Clinical characteristics including sex, pathological diagnosis, performance score, radiological characteristics of brain metastasis, and local treatments for brain metastasis were not significantly different between the two groups. However, the thicker TMT group was significantly younger than the thinner TMT group (mean age, 61.7, range 33–90 years versus 66.9, range 36–90 years, *p* < 0.001). The baseline characteristics of these patients at the time of diagnosis for brain metastasis are shown in Table 1.

### 3.2. Temporal Muscle Thickness and Overall Survival

**Figure 2:**
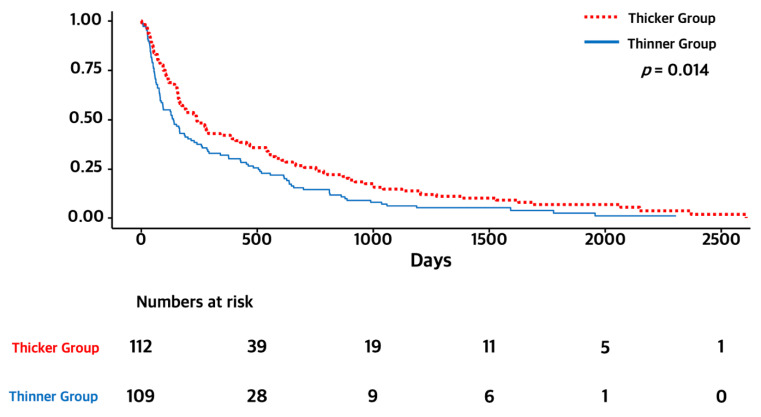
Kaplan–Meier survival graphs of OS for thicker and thinner TMT groups.

The overall survival and median duration of follow-up were 5.0 months (range: 0–85 months). The thicker TMT group showed longer survival than the thinner TMT group on log-rank test (7.0 months versus 4.0 months, *p* = 0.014). The Kaplan–Meier survival curves of OS for each group are presented in Figure 2. We performed univariate and multivariate Cox regression analyses for OS with established prognostic factors to validate whether TMT was an independent prognostic factor. In multivariate analysis for OS, a thicker TMT was associated with longer OS (HR 0.73 CI 0.55–0.96, *p* = 0.022). In addition, male sex and older age (≥65 years) were associated with shorter OS (male sex, HR 1.56 CI 1.18–2.06, *p* = 0.020; older age, HR 2.09 CI 1.57–2.79, *p* < 0.001), while performance status and any local treatment for brain metastasis were not associated with OS. The detailed results are presented in Table 2.

### 3.3. Subgroup Analysis of Elderly Patients (≥65 Years)

**Figure 3:**
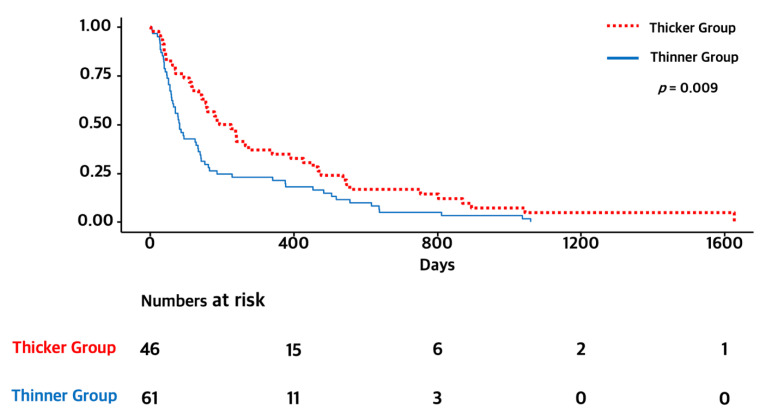
Kaplan–Meier survival graphs of OS for thicker and thinner TMT groups in the elderly subgroup.

**Figure 4:**
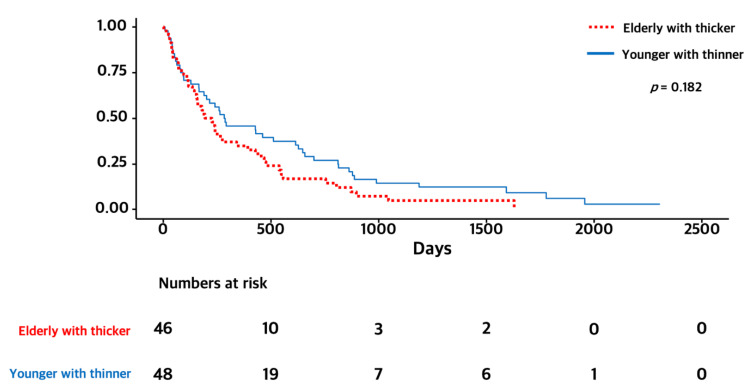
Kaplan–Meier survival graphs of OS for elderly patients with thicker TMT and younger patients with thinner TMT.

As age was a strong prognostic factor and elderly people showed a poor prognosis, we performed subgroup analysis. In a subgroup of elderly patients (≥65 years), the OS of the thicker TMT group was significantly longer than that of the thinner group (6.5 months versus 2.5 months, *p* = 0.009). We also compared elderly patients with thicker TMT and younger patients with thinner TMT. There were no significant differences between the two groups (6.5 months versus 9.5 months, *p* = 0.182). The Kaplan–Meier survival curves of OS for each subgroup are presented in Figure 3 and Figure 4.

## 4. Discussions

In this study, we used a sex-based median cut-off value for TMT and enrolled 112 patients classified in the “thicker group” and 109 patients in the “thinner TMT group”. Our findings showed that the thicker TMT group exhibited longer survival than the thinner group (6.5 months versus 2.5 months, *p* = 0.009). and thicker TMT was an independent prognostic factor (HR 0.73 CI 0.56–0.96, *p* = 0.024). Furthermore, male sex and older age (≥65 years) which are well known as poor prognostic factors in brain tumors were found to be independent prognostic factors. Furthermore, identical results were demonstrated in subgroup analysis of elderly patients. Interestingly, in our subgroup analysis of elderly patients (≥65 years), the difference in OS between the thicker and thinner groups was more prominent than observed in the population as a whole (6.5 months versus 2.5 months, *p* = 0.009). We also compared elderly patients with thicker TMT and younger patients with thinner TMT, and there were no significant differences between the two groups (6.5 months versus 9.5 months, *p* = 0.182). This may imply that a novel biomarker representing for biological age are needed in patients with brain metastasis. More aggressive treatments could be considered for patients with older chronological age but younger biological age. Taken together, our study added more evidence to support that TMT could be an independent prognostic marker for patients with brain metastasis.

Furtner et al. played a pioneering role in the relationship between sarcopenia and the prognosis of brain tumors. The relationship was revealed in many types of brain cancer, such as glioblastoma (GBM), melanoma, and metastatic tumors [10,14,15,16,17,19]. In 2019, they conducted translational imaging analysis in the EORTC 26101 trial in GBM and concluded that reduced TMT is an independent negative prognostic parameter in patients with progressive GBM and may be useful for stratifying patients for therapeutic interventions or clinical trials [16].

As patients with brain metastasis experience variable responses to treatment and survival rates due to disease heterogeneity, numerous studies have tried to classify patients with brain metastasis as a prediction of their prognosis. Since recursive partitioning analysis (RPA) was first developed by the Radiation Therapy Oncology Group (RTOG) in 1997 [20], several different scores and grading systems have been introduced [21]. Graded Prognostic Assessment (GPA) was introduced as a new prognostic index for patients with brain metastasis by Sperduto et al. in 2008 [22]. Since then, disease-specific GPA (ds-GPA) was developed to reflect the diversity of brain metastasis according to various primary cancers [23]. More recently, molecular markers were added, EFGR and ALK alteration have been used as a factor for grading lung cancer patients with brain metastasis (Lung-molGPA) [3]. This scoring system can also serve as criteria for clinical trials in terms of inclusion/exclusion [21].

As a measurement of TMT is very simple and reproducible, we suggest that TMT could be added to scoring systems to predict the prognosis of patients with brain metastasis. Considering that local treatment modalities vary in patients with brain metastasis and choosing an appropriate modality is controversial, we also suggest that TMT could be useful in selecting treatment modalities. Further investigation of prognosis prediction grading systems using TMT is warranted.

Our study has several limitations. First, due to its retrospective nature, there could be several biases related to the collection and interpretation of medical records data. Second, TMT varied according to sex and age. Although our multivariate analysis including sex and age showed that TMT was an independent prognostic marker, there could also be a bias because our findings showed that TMT was correlated with age and sex. Third, molecular analysis of EGFR and ALK was not performed in all patients. The lack of information on molecular markers could have biased our findings. Lastly, TMT has a relatively small diameter to be measured, they also could be inter-observer biases, although previous studies suggested that the measurement of TMT was not significantly different between the observers [24].

## 5. Conclusions

Our findings suggest that TMT can be a useful biomarker for OS in NSCLC patients with brain metastasis. Further investigation of the development or modification of prognosis prediction systems using TMT is warranted.

## Figures and Tables

**Table 1:** Baseline characteristics.

Variates	Thicker TMT Group (*n* = 112)	Thinner TMT Group (*n* = 109)	*p*-Value
Male sex, *n* (%)	67 (59.8)	66 (60.6)	>0.999
Age, years, *n* (range)	61.7 (33–90)	66.9 (36–90)	<0.001
≥65 years, *n* (%)	46 (41.1)	61 (56.0)	0.038
TMT, mm (range)			
Male	9.6 mm (7.7–14.8)	6.0 mm (2.4–7.6)	<0.001
Female	8.7 mm (7.0–11.7)	5.7 mm (2.4–6.9)	<0.001
Pathological diagnosis, *n* (%)			0.511
Adenocarcinoma	88 (78.6)	78 (71.6)	
Squamous cell carcinoma	13 (11.6)	17 (15.6)	
Large cell carcinoma	2 (1.8)	1 (0.9)	
Sarcomatoid carcinoma	1 (0.9)	4 (3.7)	
Others	8 (7.1)	9 (8.3)	
Number of brain metastases, *n* (%)			0.286
1	29 (25.9)	35 (32.1)	
2–4	36 (32.1)	31 (28.4)	
5	47 (42.0)	43 (39.5)	
Extracranial metastases, *n* (%)	55 (49.1)	49 (45.0)	0.629
ECOG, *n* (%)			0.358
0–1	65 (58.0)	62 (56.9)	
2	47 (42.0)	47 (43.1)	
Radiotherapy, *n* (%)	82 (73.2)	72 (66.1)	0.312
Radiosurgery, *n* (%)	15 (13.4)	7 (6.4)	0.132
Surgical resection, *n* (%)	12 (10.7)	6 (5.5)	0.242
EGFR mutation, *n* (%)			0.107
Yes	52 (46.4%)	37 (33.9%)	
No	43 (38.4%)	46 (42.2%)	
Unknown	17 (15.2%)	26 (23.9%)	
ALK mutation, *n* (%)			0.681
Yes	2 (1.8%)	1 (0.9%)	
No	58 (51.8%)	52 (47.7%)	
Unknown	108 (48.9%)	56 (51.4%)	

ALK, anaplastic lymphoma kinase; ECOG, Eastern Cooperative Oncology Group; EGFR, epidermal growth factor receptor; TMT, temporal muscle thickness.

**Table 2:** Univariate and multivariate Cox regression analysis of overall survival.

	Univariate Analysis		Multivariate Analysis	
Variates	Hazard Ratio (95% CI)	*p* Value	Hazard Ratio (95% CI)	*p* Value
Sex (male versus female)	1.43 [1.08, 1.88]	0.012	1.56 [1.18, 2.06]	0.020
Age (≥65 versus <65)	1.98 [1.49, 2.63]	<0.001	2.09 [1.57, 2.79]	<0.001
TMT (thicker versus <thinner)	0.71 [0.54, 0.93]	0.014	0.73 [0.55, 0.96]	0.022
Extracranial metastases (presence versus absence)	0.87 [0.66, 1.14]	0.300		
Number of brain metastases (single versus multiple)	1.04 [0.77, 1.41]	0.784		
ECOG (0 or 1versus ≥2)	0.79 [0.60, 1.04]	0.096	0.78 [0.59, 1.02]	0.071

CI, confidence interval; ECOG, Eastern Cooperative Oncology Group; TMT, temporal muscle thickness.

## Data Availability

Data are available only on request due to privacy/ethical restrictions.
